# NLRP3 Inflammasome: Key Mediator of Neuroinflammation in Murine Japanese Encephalitis

**DOI:** 10.1371/journal.pone.0032270

**Published:** 2012-02-29

**Authors:** Deepak Kumar Kaushik, Malvika Gupta, Kanhaiya Lal Kumawat, Anirban Basu

**Affiliations:** National Brain Research Centre, Manesar, Haryana, India; University of Texas Medical Branch, United States of America

## Abstract

**Background:**

Japanese Encephalitis virus (JEV) is a common cause of acute and epidemic viral encephalitis. JEV infection is associated with microglial activation resulting in the production of pro-inflammatory cytokines including Interleukin-1 β (IL-1β) and Interleukin-18 (IL-18). The Pattern Recognition Receptors (PRRs) and the underlying mechanism by which microglia identify the viral particle leading to the production of these cytokines is unknown.

**Methodology/Principal Findings:**

For our studies, we have used murine model of JEV infection as well as BV-2 mouse microglia cell line. In this study, we have identified a signalling pathway which leads to the activation of caspase-1 as the key enzyme responsible for the maturation of both IL-1β and IL-18 in NACHT, LRR and PYD domains-containing protein-3 (NLRP3) dependent manner. Depletion of NLRP3 results in the reduction of caspase-1 activity and subsequent production of these cytokines.

**Conclusion/Significance:**

Our results identify a mechanism mediated by Reactive Oxygen Species (ROS) production and potassium efflux as the two danger signals that link JEV infection to caspase-1 activation resulting in subsequent IL-1β and IL-18 maturation.

## Introduction

Microglia are the resident macrophages of the Central Nervous System (CNS), which are monocytic in origin and migrate to the CNS during early embryonic development [1], [2]. Microglia are the primary cells that drive the inflammatory response within this organization of the body during neurodegenerative diseases as well as other pathologies of the brain. Viral encephalitis is the most common brain pathology and it is caused by a number of viruses that invade this organ [3]. Japanese Encephalitis Virus (JEV), a single-stranded RNA (ssRNA) virus, is one of the common arboviruses that causes severe brain pathology [4] manifesting with fever, headache, vomiting and signs of meningeal irritation resulting in high mortality [5] and is responsible for a majority of encephalitis cases in Asiatic region [6]. During infection in the CNS, JEV initiates a potent inflammatory response including microglial activation which subsequently results in the production of several pro- and anti-inflammatory cytokines including IL-1β and IL-18 [7], [8]. Both IL-1β and IL-18 play an important role in fever, septic shock and inflammatory diseases [9]. In CNS, these cytokines are detrimental for neuronal health and an uncontrolled regulation of their secretion could lead to bystander neuronal damage [7] leading to neurological sequelae associated with JEV infection. Understanding the pathway leading to the production of IL-1β and IL-18 can be valuable for designing therapeutic approaches to control the secretion of these cytokines. However, the machinery responsible for IL-1β and IL-18 production by microglia during JEV infection is not understood and therefore it is critical to comprehend the mechanisms regulating the production of these cytokines in response to JEV infection.

Like their peripheral counterparts, microglia express several PRRs for the identification of viruses [3], [10]. Toll-like receptors (TLRs) recognize microbial as well as non microbial products including lipopolysaccharide (LPS), urate crystals, collagen [11] as well as viral pathogens [12], [13] on the membrane surfaces. Other PRRs such as helicase-domain-containing antiviral proteins, Retinoic acid inducible gene-I (RIG-I) and Melanoma differentiation-associated gene 5 (MDA5) as well as Nod-like receptors (NLRs) identify the pathogen intracellularly [10], [14]–[16]. Several NLRs have been reported to be involved in recognition of viruses by the cells of monocytic lineage [17]. NLRs, in addition to identifying pathogen associated molecular patterns (PAMPs), also necessitate the presence of other host derived danger associated molecular patterns (DAMPs) [18]–[21]. However, information on the role of NLRs during JEV pathology is lacking. Among all the NLRs reported, NLRP3 is the most studied and best characterized intracellular receptor [22]. Upon recognition of a pathogen, NLRP3 interacts with an adaptor molecule, Apoptosis-associated speck-like protein containing a CARD (ASC). ASC in turn interacts specifically with procaspase-1 via their Caspase Recruitment Domain (CARD) [23] thereby activating Caspase-1 [24]. This complex termed as inflammasome further processes and activates pro-IL-1β and pro-IL-18 to their mature forms, which then mediate numerous innate and adaptive immune responses upon secretion [25].

In addition to their involvement in several inflammatory [26] and neurodegenerative diseases [27], NLRP3 is engaged in mediating inflammation during viral infections. It is involved in the identification of double-stranded DNA viruses like adenovirus [20], [28] and Varicella Zoster Virus [29] as well as several ssRNA viruses including Sendai virus, Influenza viruses [19], [25], encephalomyocarditis virus and vesicular stomatitis virus [30]. Recent studies have shown that NLRP3 is not only important for adaptive immune response against Influenza virus [21], [25], it also stimulated the secretion of IL-1β and IL-18 via its M2 protein in primed macrophages and dendritic cells [31]. Thomas et al previously made similar observations that innate immunity was compromised in Nlrp3 (−/−) and Casp (−/−) mice during Influenza A virus infection [32] which indicates that NLRP3 plays an important role in the identification of ssRNA viruses. However, the involvement of NLRP3 in mediating the production of IL-1β and IL-18 in response to JEV infection is not recognized. The goal of this research was to understand the involvement of NLRP3 in processing and maturation of caspase-1 and subsequent production of IL-1β and IL-18 in response to JEV and also to unravel the underlying molecular mechanisms involved in the production of these pro-inflammatory cytokines in microglial cells.

## Results

### JEV induces IL-1β and IL-18 production *in vivo*

**Figure 1:**
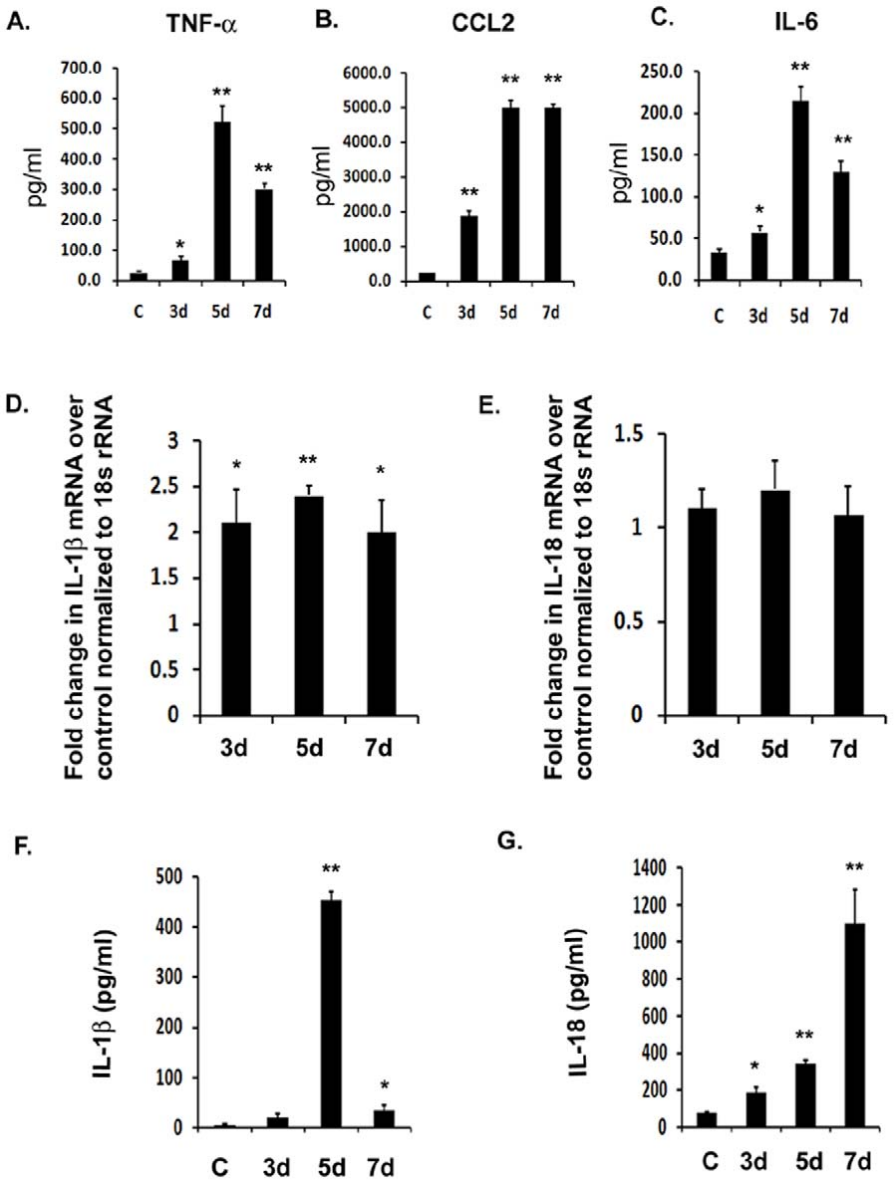
JEV induces the production of IL-1β and IL-18 *in vivo*. Infection was carried out in BALB/c mice with 5×10^5^ PFU of JEV Intravenously. Brain samples were isolated from mock-infected control (C) as well JEV infected mice after 3 d, 5 d and 7 d post infection. (**A–C**) Cytokine levels were estimated using CBA. The graphs represent the levels of pro-inflammatory cytokines, TNF-α, CCL2 and IL-6 in pg/ml from protein homogenates isolated from infected and mock-infected mice brain (**D–E**) qRT-PCR analysis was carried out from total RNA isolated from mock-infected and JEV infected mice brains on all the time points and levels of IL-1β as well as IL-18 mRNA were measured. Graphs represent fold change in mRNA values with respect to mock-infected control normalized to 18 s rRNA internal control. (**F–G**) ELISA study was carried out to measure the levels of mature IL-1β and IL-18 cytokine from JEV infected as well as uninfected brain samples. Graphs represent the cytokine levels in pg/ml in mock-infected control as well as infected brain samples. Data represent mean ± SEM of 5 animals in each group. Statistical differences were evaluated using one way ANOVA with Bonferroni's post hoc test. *, **, Statistical differences in comparison to mock-infected control values (* p<0.05; ** p<0.01).

We wanted to know the magnitude of IL-1β and IL-18 secretion in adult mice brain during JEV infection. Therefore, we infected 6–8 week old BALB/c mice with 5×10**^5^** Plaque Forming Units (PFU) for different time points. At the very onset, we measured the levels of pro-inflammatory cytokines from JEV infected as well as mock-infected mice brain (C) to confirm the establishment of infection. We observed that all the three cytokines, tumor necrosis factor- α (TNF-α), Chemokine (C-C motif) ligand 2 (CCL2) also known as macrophage chemoattractant protein-1 (MCP-1) and interleukin-6 (IL-6) were up regulated by several folds in infected mice with respect to mock-infected animals (Fig. 1A). There was a significant and gradual increase in TNF-α by 2.5-fold, 20-fold and 10-fold on 3 d, 5 d and 7 d post infection (d.p.i) respectively. Similarly, there was a significant increase in CCL2 by more than 7-fold after 3 d of infection and more than 20-fold increase on 5 and 7 d.p.i. respectively (Fig. 1B). The levels of IL-6 also significantly increased by more than 1.5-fold after 3 d.p.i. as well as by 6-fold and 4-folds on after 5 and 7 days post infection respectively, suggesting a robust inflammation upon JEV infection in mice brain (Fig. 1C). We then carried out quantitative Real Time-PCR (qRT-PCR) for IL-1β and IL-18 mRNA and observed a significant increase in IL-1β mRNA levels at all the time points with levels increasing by more than 2-folds on 3 and 5 d.p.i. upon JEV infection (Fig. 1D). However, qRT-PCR analysis of IL-18 mRNA expression suggested that there was no significant increase in their levels even after 7 days of infection (Fig. 1E). This discrepancy would have affected the levels of these cytokines at protein levels. We therefore wanted to estimate their protein levels.

To determine the changes in protein levels of IL-1β and IL-18, we performed ELISA to measure the secretion of active IL-1β and IL-18 proteins in mice brain upon JEV infection. We observed a significant increase in IL-1β levels in mice brain upon JEV infection in a time dependent manner (Fig. 1F). There was a maximum increase in IL-1β levels by 110-folds which was observed on 5 d post infection (Fig. 1F). In contrast to IL-18 mRNA levels, mature IL-18 protein levels significantly increased in mice infected with JEV in a time-dependent manner (Fig. 1G). In this case, the maximum increase was observed on 7 d.p.i. where the IL-18 levels increased by more than 14-folds over that of mock-infected control group (Fig. 1G). These observations reflect that there is a significant upregulation of IL-1β and IL-18 in mice brain upon JEV infection. The differences in IL-18 mRNA levels, however did not correlate with that of its protein levels in the current time point analysis.

### JEV induces robust inflammation *in vitro* along with IL-1β and IL-18 production

**Figure 2:**
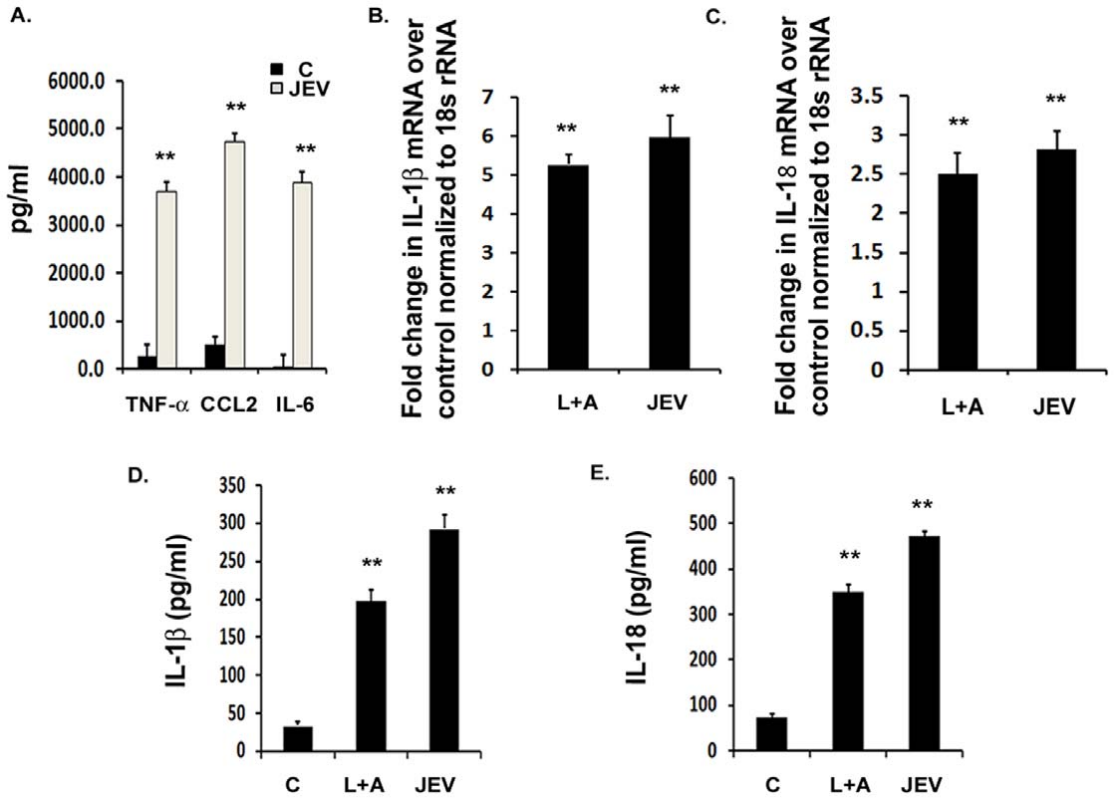
JEV induces the production of IL-1β and IL-18 *in vitro*. BV-2 microglial cells were infected with 5 MOI of JEV for different time points. LPS+ATP condition was used as a positive control for qRT-PCR, ELISA and caspase-1 activity studies. (**A**) Cytokine analysis from mock-infected control and JEV infected BV-2 cells was carried out after 6 h of JEV infection using cytokine bead array. Graphs represent the levels of pro-inflammatory cytokines, TNF- α, CCL2 and IL-6 after JEV infection with respect to mock-infected control condition. (**B–C**) qRT-PCR studies on total RNA isolated from uninfected cells as well as on BV-2 cells infected with JEV for 3 h. IL-1β and IL-18 mRNA levels are represented in terms of fold change with respect to mock-infected control normalized to 18 s rRNA internal control. (**D–E**) Levels of mature IL-1β and IL-18 cytokines upon JEV infection with respect to mock-infected control condition were measured by ELISA. Graphs represent the fold change values in JEV infected cells with respect to mock-infected control condition. Data represent mean ± SEM from 3 independent experiments performed in duplicate. Statistical differences were evaluated using one way ANOVA with Bonferroni's post hoc test. *, **, Statistical differences in comparison to mock-infected control values (* p<0.05; ** p<0.01).

In order to validate the results obtained from mice brain and to identify a mechanism for the secretion of inflammatory cytokines, IL-1β and IL-18, we infected BV-2 mouse microglial cell line with 5 MOI dose of JEV. JEV replicates very well in BV-2 cells and can productively infect these cells without any morphological alterations [33]. BV-2 cells respond to JEV by secreting several pro-inflammatory cytokines and chemokines [8]. Therefore, in order to confirm the onset of inflammation, we measured the levels of pro-inflammatory cytokines like TNF-α, CCL2 and IL-6 and found that their levels increased significantly over mock-infected control condition within 6 h of JEV infection. There was more than 13-fold increase in TNF-α, 9-fold in CCL2 and approximately 90-fold increase in IL-6 levels (Fig. 2A) upon JEV infection with respect to mock-infected condition. This confirmed the activation of microglia in response to the virus and was followed by qRT-PCR analysis for IL-1β and IL-18 mRNA expression. Infection with JEV for 3 h resulted in increased IL-1β and IL-18 mRNA levels in infected cells with respect to mock-infected condition. We observed a significant 5-fold increase in IL-1β mRNA (Fig. 2B) and more than 2.5-fold increase in IL-18 mRNA (Fig. 2C) upon JEV infection with respect to mock-infected control. LPS+ATP is known to stimulate the production of the transcripts of these cytokines, therefore, we used it as a positive control.

In order to estimate the IL-1β and IL-18 protein levels, we carried out ELISA from the total protein isolated from BV-2 cells. We observed a significant 8-fold increase in IL-1β levels in JEV condition over that of mock-infected control and also observed a significant increase in LPS+ATP conditions (Fig. 2D). Similarly, IL-18 cytokine levels were increased by more than 6-fold in JEV condition along with a significant increase in LPS+ATP condition with respect to mock-infected control (Fig. 2E). These results suggest that both IL-1β and IL-18 transcript as well as mature protein levels get up-regulated in BV-2 microglial cells upon JEV infection.

### JEV induces caspase-1 activation *in vivo* and *in vitro*

**Figure 3:**
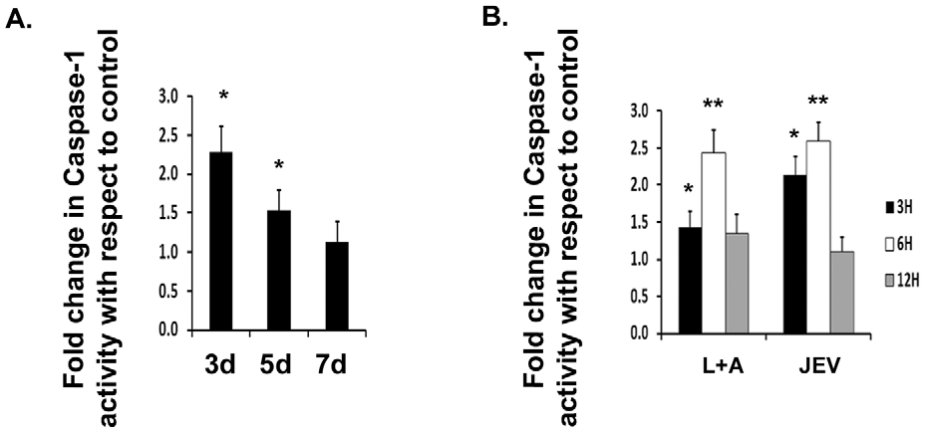
JEV induces caspase-1 activation *in vivo* and *in vitro*. Infection was carried out in BALB/c mice with 5×10^5^ PFU of JEV Intravenously. Brain samples were isolated from mock-infected control (C) as well JEV infected mice after 3 d, 5 d and 7 d post infection for estimation of caspase-1 activity. (**A**) Caspase-1 activity is represented as fold change in caspase-1 activity in JEV infected brain with respect to mock-infected brain. Data represent mean ± SEM of 5 animals in each group. (**B**) Caspase-1 activity was measured in BV-2 cells infected with 5 MOI of JEV after 3 h, 6 h and 12 h of JEV infection over that of mock-infected condition. Caspase-1 activity is represented as fold change with respect to mock-infected condition. Data represent mean ± SEM from 3 independent experiments performed in duplicate. Statistical differences were evaluated using one way ANOVA with Bonferroni's post hoc test. *, **, Statistical differences in comparison to mock-infected control values (* p<0.05; ** p<0.01).

As mentioned earlier, caspase-1 is synthesized as an inactive preprotein [24] which is converted to the active form after cleavage of its C-terminus [34]. Production of mature IL-1β and IL-18 requires the activation of caspase-1 enzyme which cleaves immature pro- IL-1β and pro-IL-18 in response to inflammatory agents [19]. We therefore wanted to know if activation of caspase-1 occurred upon JEV infection both in BV-2 cells as well as in mice brain. Using caspase-1 activity assay, we observed a significant caspase-1 activity in JEV infected mice brain over that of mock-infected control brain. There was a significant 2-fold increase in caspase-1 activation on 3 d post infection. Even after 5 days of infection, we observed more than 1.5-folds increase in caspase-1 activity over that of mock-infected mice brain (Fig. 3A) and the levels gradually decreased thereafter. We did not observe any significant change in caspase-1 activity in mice brain after 7 days of JEV infection. Similarly, upon JEV infection *in vitro*, we observed an increase in caspase-1 activity. We observed a time dependent increase in its activity by 3 h and 6 h post infection withmaximum activity being observed after 6 h of treatment by both LPS+ATP as well JEV (Fig. 3B). To the best of our knowledge, this is the first report of caspase-1 activation during JEV infection in mice brain and microglial cells.

### Replicating JEV and not the host derived factors are responsible for caspase-1 activity and subsequent production of IL-1β and IL-18

In order to rule out the possibility of caspase-1 activation by host derived factors, we used mice brain homogenate from control mice (Ctrl) to treat BV-2 cells along with homogenates from JEV treated mice (JEV) and carried out the caspase-1 activity assay and measured IL-1β and IL-18 in these cells. We also treated BV-2 cells with UV irradiated homogenates from JEV treated mice brain (UV-JEV) in order to confirm the role played by replication competent JEV in mediating the cytokine production. We found that UV irradiation significantly affected the plaque formation by JEV (Fig S1A) suggesting the altered replication efficiency of the virus. We observed no significant activity of caspase-1 in either Ctrl or UV-JEV conditions with respect to JEV condition (Fig S1B). Moreover, there was a significant decrease in the levels of IL-1β (Fig S1C) and IL-18 (Fig S1D) in both, Ctrl and UV-JEV condition when compared with JEV infected condition. These findings suggest that replicating JE virus and not the replication incompetent virus activates inflammasome complex in order to initiate caspase-1 activity and subsequent production of IL-1β and IL-18. In addition, these observations also suggest that host derived factors from mock-infected mice brain homogenate do not active this complex.

### Caspase-1 activity is required for production of IL-1β and IL-18 during JEV infection *in vitro*

**Figure 4:**
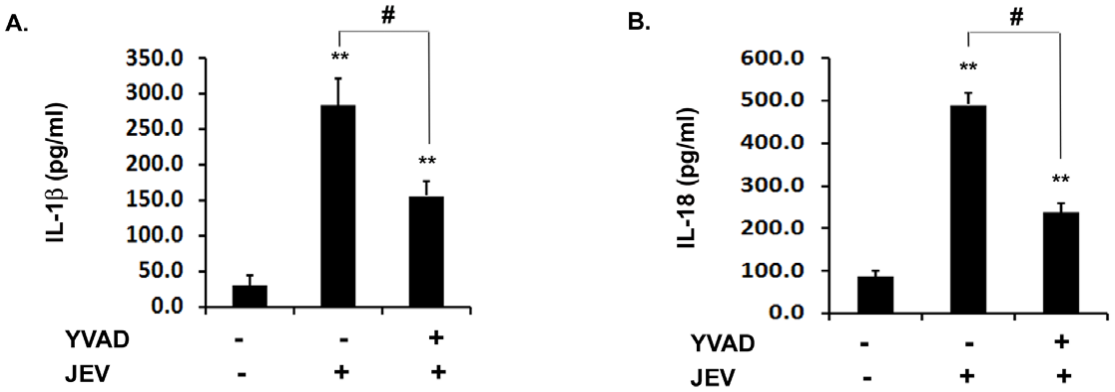
Caspase-1 activity is required for the production of IL-1β and IL-18 during JEV infection. BV-2 cells were incubated with 5 µM YVAD for 30 min to inhibit caspase-1 activity followed by JEV infection. (**A–B**) ELISA for IL-1β and IL-18 was carried out in JEV infected BV-2 cells upon caspase-1 inhibition. The cytokine levels were then measured using ELISA and the values are represented in pg/ml. Data represent mean ± SEM from 3 independent experiments performed in duplicate. Statistical differences were evaluated using one way ANOVA with Bonferroni's post hoc test. **, Statistical difference in comparison to mock-infected control values (** p<0.01) and #, Statistical difference with respect to JEV infected condition (p<0.01).

In order to find out whether an active caspase-1 is required for the maturation of IL-1β and IL-18 to their active forms in BV-2 cells upon JEV infection, we inhibited caspase-1 activity using the caspase-1 inhibitor Z-YVAD-FMKYVAD (YVAD) and then the cells were infected with JEV for 6 h as we had previously observed a significant increase in caspase-1 activity at this time point. JEV infection increased IL-1β and IL-18 levels significantly at this time point (Fig. 4A & 4B). However, the inhibition of caspase-1 activity (data not shown) resulted in significant decrease in both IL-1β (Fig. 4A) and IL-18 (Fig. 4B) levels by more than 2-folds. This finding suggests that caspase-1 is crucial for the maturation of IL-1β and IL-18 during JEV infection in mouse microglia.

### Knockdown of NLRP3 decreases caspase-1 activity and subsequent production of IL-1β and IL-18 upon JEV infection

**Figure 5:**
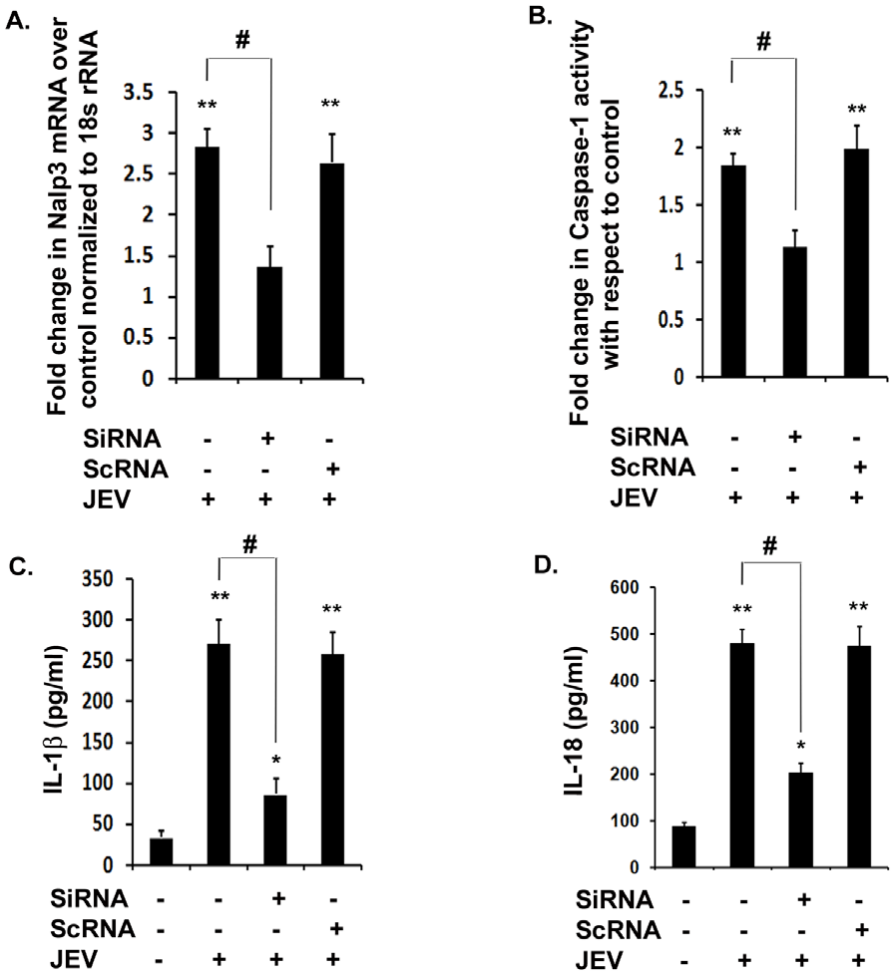
NLRP3 is critical for caspase-1 activity as well as IL-1β and IL-18 production. Transient knockdown of NLRP3 using 100 nM of NLRP3 SiRNA was carried out in BV-2 microglial cells. Scrambed RNA (ScRNA) was used as a transfection control. This was followed by virus infection with 5 MOI dose for 6 h. (**A**) NLRP3 mRNA levels were measured by qRT-PCR from total RNA isolated from JEV infected as well as uninfected BV-2 cells. The graph represent a fold change in NLRP3 mRNA with respect to mock-infected control normalised to 18 s rRNA internal control. (**B**) Caspase-1 activity was also measured in NLRP3 knockdown condition upon JEV infection. Graph represents fold change in caspase-1 activity in different conditions with respect to mock-infected control. (**C–D**) In order to measure IL-1β and IL-18 production, ELISA was then carried out in NLRP3 knockdown (SiRNA+JEV) condition with respect to JEV infected sample. Graph represents cytokine levels in pg/ml. Data represent mean ± SEM from 3 independent experiments performed in duplicate. Statistical differences were evaluated using one way ANOVA with Bonferroni's post hoc test. *, **, Statistical difference in comparison to mock-infected control values (* p<0.05, ** p<0.01) and #, Statistical difference with respect to JEV infected condition (p<0.01).

NLRP3 is one of the key inflammasome molecules implicated in identification of ssRNA and double-stranded DNA viruses [19]. In order to determine whether NLRP3 is required for caspase-1 maturation upon JEV infection, we first carried out SiRNA mediated knockdown of NLRP3. We observed that in JEV infected BV-2 cells, there was more than 2.5-fold induction in NLRP3 mRNA levels with respect to mock-infected control condition (Fig. 5A). Upon JEV infection, NLRP3 SiRNA treatment(SiRNA+JEV) resulted in a significant decrease in NLRP3 mRNA levels by about 50% confirming a substantial knockdown of NLRP3 transcript while there was no change observed in the cells transfected with scrambled RNA upon JEV infection (ScRNA+JEV) with respect to JEV alone treatment (Fig. 5A). In order to confirm that NLRP3 inflammasome is required for caspase-1 maturation and its activity, we analyzed caspase-1 activity in NLRP3 knockdown conditions upon JEV infection. As hypothesized, we observed a significant reduction of caspase-1 activity by 1.8-folds in SiRNA+JEV condition compared to JEV alone condition (Fig. 5B). There was no change in caspase-1 activity in ScRNA+JEV condition with respect to JEV alone condition. We then carried out ELISA to measure IL-1β and IL-18 expression levels under different conditions. We observed a 3-fold reduction in IL-1β levels in SiRNA+JEV condition with respect to the JEV alone condition (Fig. 5C). Similarly, IL-18 levels were also significantly reduced by 2-folds upon JEV treatment in NLRP3 knockdown condition with respect to JEV alone treatment (Fig. 5D). This experiment is the first evidence of NLRP3 dependent IL-1β and IL-18 maturation in JEV infection. We can conclude from our findings that NLRP3 is one of the key mediators of host responses as it is crucial for the production of IL-1β and IL-18 during JEV infection.

### Generation of ROS is crucial for caspase-1 activity and subsequent IL-1β and IL-18 production during JEV infection

**Figure 6:**
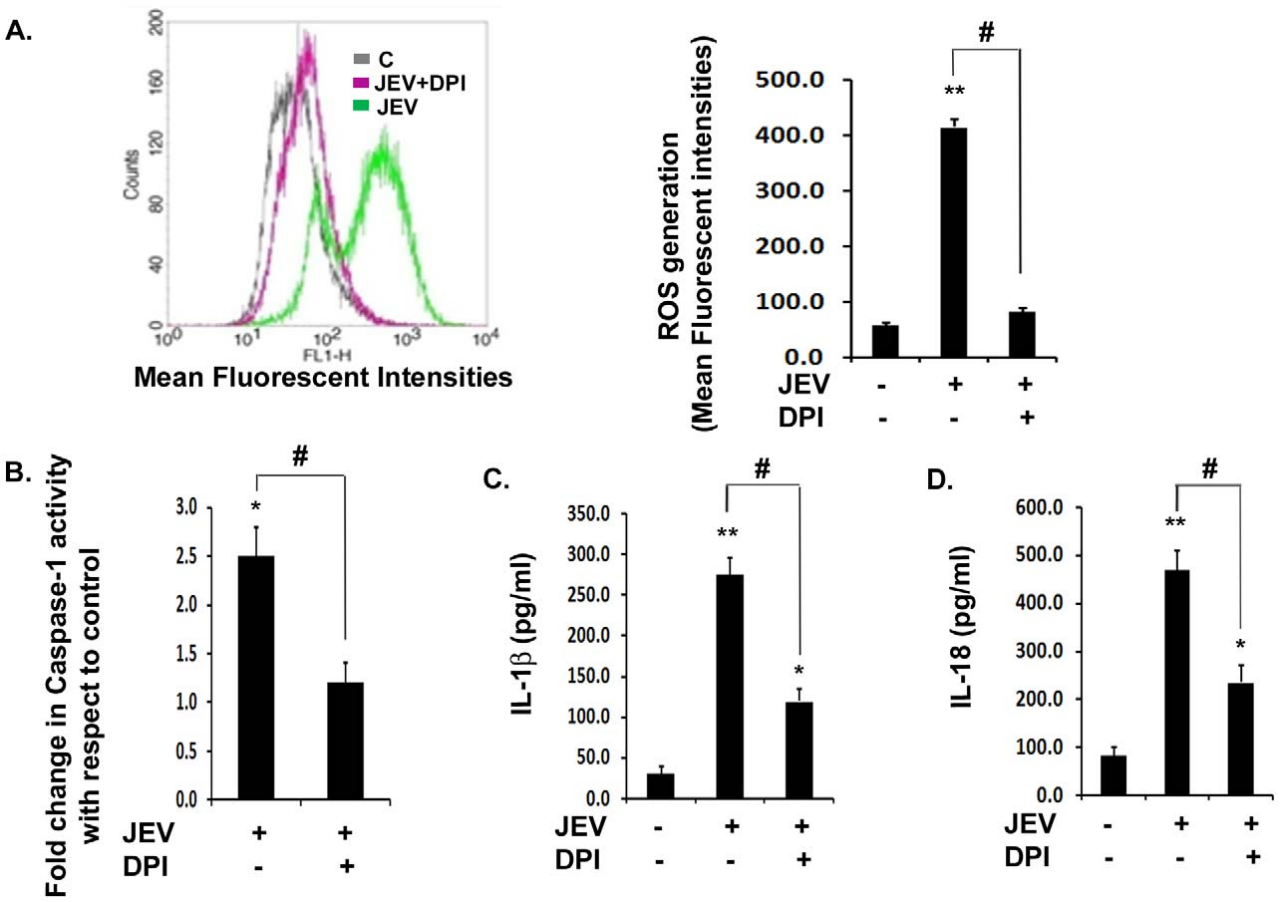
Generation of ROS is critical for caspase-1 activity and subsequent IL-1β and IL-18 maturation. BV-2 cells were incubated with 1 µM of DPI for inhibition of ROS generation. (**A**) Representative FACS plot showing intracellular ROS production, after 4 h of JEV infection and representation of the Mean Fluorescent Intensities (M.F.I) (right graph) in mock-infected control (C), JEV+DPI and JEV alone condition. (**B**) Caspase-1 activity measured after 6 h of JEV infection in presence or absence of DPI. Graph represents fold change in caspase-1 activity with respect to mock-infected control. (**C–D**) ELISA study showing the levels of mature IL-1β and IL-18 in JEV+DPI condition with respect to JEV alone infected sample. Graph represents cytokine levels in pg/ml. For Potassium efflux study, BV-2 cells were incubated with 50 mM KCl for 20 min in order to study the requirement of K**^+^** efflux for caspase-1 activity and its downstream effects. Data represent mean ± SEM from 3 independent experiments performed in duplicate. Statistical differences were evaluated using one way ANOVA with Bonferroni's post hoc test. *, **, Statistical difference in comparison to mock-infected control values (*p<0.05, ** p<0.01) and #, Statistical difference with respect to JEV infected condition (p<0.01).

Requirement of ROS as a danger signal for inflammasome complex formation has been reported by several workers [26]. We therefore wanted to test if ROS generation plays a role in inflammasome activation during JEV infection. We observed that upon JEV infection, levels of ROS increased significantly by 8-fold with respect to mock-infected control condition (Fig. 6A). However, upon addition of DPI, a potent ROS-inhibitor, there was a significant drop in ROS levels by 5-fold with respect to JEV alone condition. We then determined caspase-1 activity during viral infection upon DPI treatment. We observed that while there was 2.5-fold increase in caspase-1 activity upon JEV infection, DPI treatment in JEV infected cells resulted in a significant reduction in caspase-1 activity with respect to JEV alone treatment (Fig. 6B). This was followed by the estimation of downstream effects of caspase-1 activity by ELISA studies which revealed that both IL-1β (Fig. 6C) and IL-18 (Fig. 6D) levels were reduced significantly by more than 2-folds upon DPI treatment along with JEV infection. These findings suggest that generation of ROS during JEV infection might acts as a stress signal for the inflammasome complex formation which in turn is crucial for the production of these cytokines.

### K^+^ efflux is critical for inflammasome activity as well as IL-1β and IL-18 production during JEV infection *in vitro*

**Figure 7:**
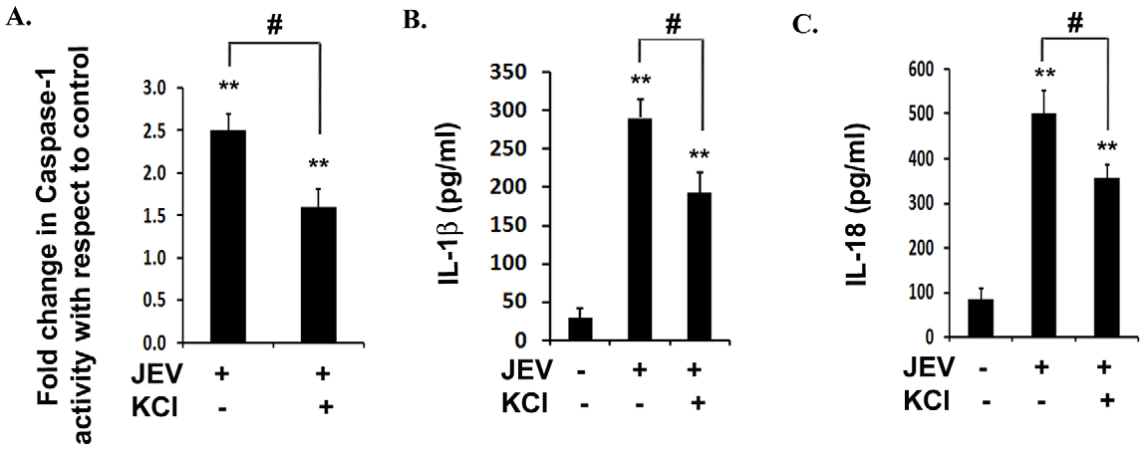
Potassium efflux is required for caspase-1 activity and subsequent inflammation upon JEV infection. (**A**) Caspase-1 activity was measured in presence of KCl upon JEV infection. Graph represents caspase-1 activity in JEV infected cells incubated with KCl with respect to untreated JEV alone condition. (**B–C**) ELISA study showing the levels of IL-1β and IL-18 in KCl treated condition upon JEV infection over that of JEV alone condition. Graph represents IL-1β and IL-18 levels in pg/ml. Data represent mean ± SEM from 3 independent experiments performed in duplicate. Statistical differences were evaluated using one way ANOVA with Bonferroni's post hoc test. *, **, Statistical difference in comparison to mock-infected control values (*p<0.05, ** p<0.01) and #, Statistical difference with respect to JEV infected condition (p<0.01).

Inflammasome assembly requires secondary signals like danger associated molecular patterns (DAMPs) which are host cell derived and are generated in response to pathogens. DAMPs like ATP are known to induce K**^+^** efflux from the cells via P2X7 receptors. Similarly, certain viruses are known to induce potassium efflux triggering inflammasome activation [20]. Therefore, we infected BV-2 cells with JEV along with or without Potassium Chloride (KCl) in the culture medium in order to maintain excessive extracellular potassium which will act as an inhibitor for K**^+^** efflux due to reversal of the concentration gradient. Interestingly, in the presence of KCl, we observed more than 2-fold reduction in caspase-1 activity with respect to JEV alone condition (Fig. 7A). This finding was further supported by significant reductions in IL-1β and IL-18 levels in presence of excess extracellular potassium (Fig. 7B and 7C). Although, we did not observe a complete abrogation of these cytokines upon KCl treatment, these findings suggest that NLRP3 inflammasome activation may require potassium efflux as an additional danger signal upon JEV infection.

## Discussion

Our lab has previously shown that microglia respond to JEV infection by secreting the pro-inflammatory IL-1β and IL-18 in addition to other proinflammatory cytokines [7], [35]. IL-1β is a multifunctional cytokine with important roles in acute and chronic inflammation [36], [37] which can trigger signal transduction pathways resulting in synthesis of additional pro-inflammatory cytokines, chemokines and acute phase proteins thereby causing fever, and hypotension [38]. Furthermore, over production of these cytokines is associated with bystander neuronal death during JEV infection [7]. Therefore, it is important to keep the levels of these cytokines under control and tightly regulated manner. In stark contrast to the available knowledge regarding microglial activation in response to JEV, the information regarding the machinery responsible for the production of IL-1β and IL-18 remains elusive. Our current study focused on the identification of the molecular mechanism for the production of pro-inflammatory cytokines IL-1β and IL-18 during JEV infection in microglia. Our findings demonstrated for the first time that in response to JEV infection, (i) there is an activation of caspase-1 both *in vitro* and *in vivo*, (ii) NLRP3 is the key mediator of caspase-1 activity and IL-1β and IL-18 production in microglial cell and (iii) generation of ROS and potassium efflux are the supplementary danger signals that are required by microglial cell for IL-1β and IL-18 production.

According to a recent study for the development of a nanoparticle based approach for increasing the vaccine efficacy against West Nile Virus, induction of NLRP3 inflammasome resulted in the enhanced potency of this vaccine [39]. While this finding indirectly suggested an involvement of NLRP3 in mediating innate immune response against a flaviviral species, there is no existing literature directly implicating the role of inflammasome in their infection. More recently, transcriptome analysis during JEV pathology showed an increased levels of caspase-1 along with the adaptor molecule, Pyrin-containing caspase recruitment domain or ASC at the transcript level in mice brain [40]. Based on these reports, we hypothesized that NLRP3 might be involved in mediating the inflammasome complex formation during JEV infection. Our study, therefore, focused on the role played by microglia in combating JEV infection and unravels a novel PRR for the identification of this virus. Our findings show that there is a robust inflammation in microglia upon JEV infection which is accompanied by an increased production of IL-1β and IL-18 both *in vitro* and *in vivo*. We used LPS+ATP as a positive control for the *in vitro* cytokine expression and caspase-1 activity experiments. LPS induces the production of pro-forms of these cytokines, while ATP potentiates this response by acting as a danger signal and ensuring the maturation of these cytokines resulting in an increased expression of both the caspase-1 activity as well as IL-1β and IL-18 cytokines. While we did not observe any increase in the pro-IL-18 mRNA in the JEV treated brain samples up to 7 days after infection, we observed a significant increase in IL-18 protein levels in these brain samples. It is quite likely that caspase-1 cleaves the already present IL-18 pre-protein to its mature forms in spite of no significant increase in pro-IL-18 mRNA. Indeed, we observed enhanced caspase-1 activity in mice brain as well in microglial cells upon JEV infection. The increase in caspase-1 activity by 2.5 folds seems to be physiologically important in our case as this enhancement is critical for substantial production of mature IL-1β and IL-18. Similar levels of caspase-1 activity were obtained in a study involving anakinra administration to arthritis patients where caspase-1 activity increased by 1.5 folds in absence of anakinra in the blood samples of patients with respect to healthy subjects [41] suggesting the physiological relevance of 2-fold increase in caspase-1 activity. The role of caspase-1 is important in the maturation of inflammatory cytokines as inhibition of caspase-1 activity with YVAD prior to JEV infection resulted in significant reduction of IL-1β and IL-18. Furthermore, our findings confirm that NLRP3 is a key NLR which recruits pro-caspase-1 into the inflammasome complex and subsequent cleavage of the pro-inflammatory IL-1β and IL-18 during JEV infection.

Several reports in the past have suggested that ROS generated by almost all the activators of NLRP3, plays a major role in the activation of NLRP3 inflammasome activation [26], [42], [43]. We therefore estimated ROS generation upon JEV infection and observed a significant increase in their levels upon infection. Pre-treatment of BV-2 cells with a ROS inhibitor, DPI, resulted in reduction of caspase-1 activity as well as expression of IL-1β and IL-18 cytokines. The mechanism of ROS mediated NLRP3 inflammasome activation is however, not fully understood. Initially phagosome associated NADPH oxidases were thought to be the primary sources of reactive oxygen superoxide ions upon phagocytosis of the inflammasome activators [26]. But studies in Chronic Granulomatous Disease (CGD) patients with mutations in four subunits of NADP oxidase complex show that despite the absence of ROS production, they have increased levels of caspase-1 activity and pro-inflammatory cytokine generation [44]. Infact, some recent reports suggest that mitochondria could be the main source of ROS for inflammasome activation [45], [46]. So, though in case of JEV, we show the addition of ROS inhibitor impairs the pro-inflammatory cytokine production, the source and mechanism as to how ROS activates NLRP3 inflammasome in response to JEV infection still needs to be elucidated.

Previous studies have also shown that high intracellular concentration of K**^+^** ions can potentially inhibit NLRP3 inflammasome activation [47]. It is well documented that ATP binds to P2X7 receptors on macrophages and subsequently results in association of P2X7 receptors with pannexin 1 and formation of a non-selective pore [48]. It has been previously demonstrated that Adenovirus induced NLRP3 inflammasome activation is hindered in the presence of potassium channel inhibitors like glyburide or excess KCl in the media thus inhibiting K**^+^** ion efflux [28]. In case of influenza virus, M2 protein, a proton-selective ion channel causes NLRP3 inflammasome activation by causing H**^+^** ion export from golgi complex [31]. It is also suggested that this ion channel activity may also result in imbalance of other ions like Na**^+^** and K**^+^** thus resulting in inflammasome activation. Our data suggests that NLRP3 inflammasome activation upon JEV infection requires K**^+^** ion efflux as an additional danger signal, however the exact mechanism by which K**^+^** ion efflux may be resulting is still unknown. The discovery of mitochondria as the organelle playing a major role in NLRP3 inflammasome activation leads to speculations that K**^+^** ion channels on the mitochondria may be controlling the levels of intracellular K**^+^** ions and control inflammasome activation. However, further studies are required to understand the detailed mechanism of K**^+^** efflux during JEV infection and the underlying mechanism for inflammasome aggregation.

**Figure 8:**
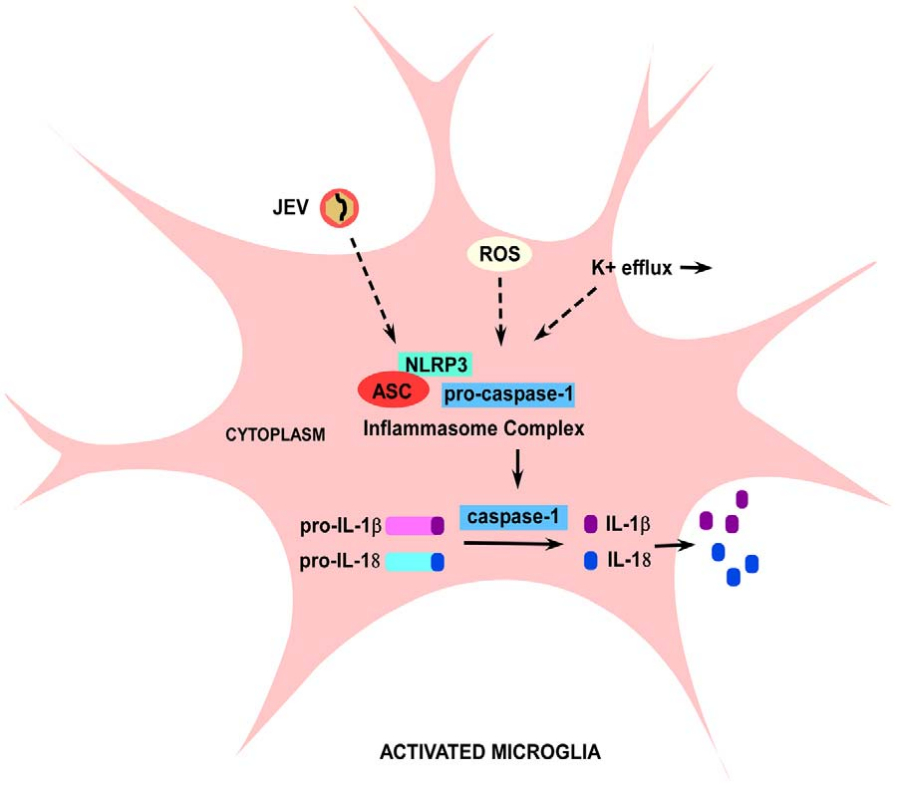
Schematic showing the signalling pathway leading to IL-1β and IL-18 production upon JEV infection in microglia. NLRP3 is the key PRR involved in the identification of JEV intracellularly in a microglia. The NLRP3 interacts with an adaptor molecule, ASC and recruits pro-caspase-1 forming a biochemical complex termed as inflammasome. In response to JEV, NLRP3 cleaves pro-caspase-1 into its active form, caspase-1. Caspase-1 then cleaves the inactive pro-forms of IL-1β and IL-18 to their mature forms which are then secreted out. ROS and K**^+^** efflux are the host derived danger signals that are critical for the formation of NLRP3 biochemical complex during JEV infection.

Our study identifies the role of NLRP3 inflammasome mediated caspase-1 activation and subsequent IL-1β and IL-18 production during JEV infection. We have also shown that replication competent JEV is crucial for activating this complex as UV treated JEV does not increase caspase-1 activity or the production of inflammatory cytokines. Although, the PAMPs associated with JEV recognized by host cells are not fully recognized, our data with UV studies suggest that inflammasome complex may identify the ssRNA genome of JEV (Fig S1) which may result in its activation. The activation of inflammasome complex in response to JEV is shown in the schematic representation (Fig. 8). We propose that upon detection of viral PAMPs, pro-caspase-1 undergoes auto-catalytic cleavage and activation, thus releasing active caspase-1. Active caspase-1 cleaves pro- IL-1β and IL-18 thus resulting in the secretion of their active forms. The generation of ROS and K^+^ ion efflux also play a major role in the activation of NLRP3 inflammasome upon JEV infection as shown in the schematic (Fig. 8). Detailed studies are required to understand in detail the pathogenic signatures of JEV which are identified by NLRP3 intracellularly. NLRP3 can be a potential target for therapeutic intervention and this information can lead to new antiviral therapies and to new insights for treating JEV infection.

## Materials and Methods

### Ethics Statement

The animals were handled in strict accordance with good animal practice guidelines defined by the Institutional Animal and Ethics Committee (IAEC) of National Brain Research Centre and the Committee for the Purpose of Control and Supervision of Experiments on Animals (CPCSEA), Ministry of Environment and Forestry, Government of India. The animal experiment protocol approval numbers were NBRC/IAEC/2007/36 and NBRC/IAEC/2008/41. All animal studies were approved by the IAEC of National Brain Research Centre.

### Virus stock, titration and Ultraviolet (UV) irradiation of JEV

JEV isolates GP78 strain are routinely maintained and propagated in BALB/c mouse pups as described earlier [49], [50]. Briefly, suckling mice (3–4 days old) were inoculated with the virus through intra-cerebral route and their brain tissue was harvested when symptoms were visible. A 10% suspension of the brain tissue was made by homogenization in the Minimal Essential Medium (MEM). It was then centrifuged at 10,000 g to remove cellular debris and filtered through a 0.22 µm sterile filter. The mouse brain derived virus was stored at −70°C in small aliquots. We used this aliquot as seed virus for further experimentation either for *in vivo* or *in vitro* systems. JEV inactivation was carried out with a UV crosslinker (UVC 500, Hoefer scientific, USA) using short-wavelength UV radiation (UVC, 254 nm) at a distance of 5 cm for 10 min on ice as described earlier [51]. Inactivation of virus was verified by plaque assay, indicating a reduction in its infectious titre by more than 10^4^ folds. Virus titration by plaque formation was carried out on the monolayers of porcine kidney (PS) cell line. PS cells were seeded in 35 mm dishes to give semi-confluent monolayers in about 18 h. Monolayers were inoculated with tenfold dilutions of virus sample made in MEM containing 1% Fetal Calf Serum (FCS) and were incubated for 1 h at 37°C with occasional shaking. The inoculum was removed by aspiration and the monolayers were overlaid with MEM containing 4% FCS, 1% low melting point agarose and a cocktail of antibiotic-antimycotic solution (Gibco) containing penicillin, streptomycin and amphotericin-B. Plates were incubated at 37°C for 3–7 days until plaques were visible. To allow counting of the plaques, the cell monolayer were stained with crystal violet after fixing the cells with 10% formaldehyde [49].

### Virus infection of animals

A previously described animal model of Japanese Encephalitis [49] was used for *in vivo* studies. Briefly, five animals per group of 6–8 weeks old BALB/c mice of either sex were injected intravenously (i.v.) with approximately 5×10**^5^** plaque forming units (PFU) of GP78 strain of JEV in 50 µl of 1× Phosphate buffer Saline (PBS) over a period of 3, 5 and 7 days. Mock-infected control animals received the same amount of 1× PBS as the experimental group. The animals of each time group were sacrificed at different time points for either protein or RNA. From the third day post infection, animals started to show symptoms of encephalitis including limb paralysis, poor pain response and whole body tremor.

### Cell Culture and reagents

Mouse microglial cell line BV-2 was originally obtained from Dr. Steve Levison, University of Medicine and Dentistry, New Jersey, USA and maintained in the lab according as previously described [52]. Briefly, BV-2 cells were grown at 37°C in Dulbecco's Modified Eagle's Medium (DMEM) supplemented with 5% Sodium Bicarbonate (NaHCO_3_), 10% Fetal Bovine Serum (FBS), penicillin at 100 units/ml and streptomycin at 100 µg/ml. The reagents related to cell culture were obtained from Sigma Aldrich, USA unless otherwise stated. At 70%–80% confluence, the cultures were treated with accutase to dislodge the microglial cells. The cells were seeded onto plastic petri dishes, and the dishes were placed in a 37°C incubator for the cells to grow.

### Infection of BV-2 cells with JEV and treatment with LPS and ATP

The mouse microglial cell line BV-2 was plated in 90 mm plastic petri dishes at the density of 1.5×10^6^ cells. After 15–18 hrs incubation in 10% DMEM, the cells were incubated in serum free media for 4–5 hrs. The cells were then adsorbed with UV-JEV and/or JEV at an MOI of 5 for 1.5 hrs. After adsorption, the unbound viruses were removed by washing gently with 1× PBS. The cells were then incubated additionally in fresh serum free media for 3 h for mRNA expression and 6 h for caspase-1 activity and cytokine secretion. Virus titration was performed with this supernatant to confirm the presence or absence of live viruses. For positive control to caspase-1 activity and secretion of IL-1β and IL-18 *in vitro*, stimulation with LPS (1 µg/ml) followed by 30 minutes exposure to 1 mM ATP was carried out. Mock-treated control cells received only 1xPBS.

### Measurement of reactive oxygen species (ROS) and treatment with KCl

We determined the effect of JEV infection on microglial cells by determining the levels of ROS as well as levels of pro-inflammatory cyto/chemokines. Intracellular ROS generation in control and treated cells was assessed using the cell permeable, non-polar H_2_O_2_ sensitive dye 5-(and-6)-chlromethyl-2′, 7′- dichlorodihydrofluorescein diacetate (CM-H_2_DCFDA) (Sigma Aldrich) as described previously [53], [54]. The extent to which H_2_O_2_ is generated is defined as the extent of ROS generation here. In order to understand the role of ROS formation in the assembly of inflammasome, ROS inhibitor, 1 µM diphenyleneiodonium chloride (DPI) (Sigma Aldrich,USA) was used. Briefly, BV-2 cells were incubated for 4 h with 1 uM DPI (Sigma Aldrich,USA) or 5 µM N-Acetyl Cysteine (NAC) (data not shown) followed by mock and JEV infection for 1.5 h. This was further followed with incubation in serum free media for 4 h. Upon treatment, the cells were further treated with H_2_DCFDA (5 µM) for 1 h at 37**°**C, washed twice with 1× PBS, and fluorescent intensity of the cells was measured using CellQuest Pro software on FACS Calibur Flowcytometer (BD, Biosciences; excitation, 488 nm; emission, 530 nm). For K**^+^** efflux experiment, DMEM media was replaced with Opti-MEM media and the cells were incubated for 20 minutes in the presence and absence of 50 mM potassium chloride. Following the incubation with KCl, the cells were adsorbed with JEV for 1.5 h at 37°C and further incubated in serum free media for 5 hrs. The protein from the total cell extracts was used to study caspase-1 activation and release of mature form of IL-1β and IL-18 by ELISA.

### ELISA for IL-1β and IL-18

ELISAs for mouse IL-1β and IL-18 were performed on total protein isolated from treated and mock-infected control BV-2 cells as described earlier [55]. Briefly, a rat monoclonal anti-mouse IL-1β antibody (MAB401; R & D Systems, Minneapolis, MN) and goat biotinylated anti-mouse IL-1β antibody (BAF401; R & D Systems) were used as coating and sandwich antibodies, respectively. Streptavidin-conjugated HRP (GE healthcare) and Tetramethylbenzidine (TMB) Substrate (Vector Labs, CA, USA) were used for detection. The reaction was stopped by adding 50 µl of Stop Solution (2 N H**_2_**SO**_4_**). The absorbance was read at 450 nm in a microplate spectrophotometer (Biorad, Australia) and the concentrations were calculated using the IL-1β standard reference curves. The IL-18 ELISA was conducted similarly and the anti-IL-18 capture antibody (D047-3; R & D Systems) and anti-IL-18 detection (D048-6; R & D Systems) antibodies were used for primary and secondary incubation respectively. To make the standard curves, the IL-1β and IL-18 mouse recombinant proteins (R & D Systems, USA) were used.

### RNA isolation and quantitative real time PCR (qRT-PCR)

After treatments, BV-2 cells were lysed and mice brain were homogenised in Trizol reagent (Sigma Aldrich) as per the manufacturer's protocol. The RNA was isolated by phenol-chloroform method as described earlier [52] and it was quantified using spectrophotometer (GE healthcare biosciences AB, Uppsala, Sweden). Oligonucleotide primer specific for mouse NLRP3 (forward: 5′-TGC TCT TCA CTG CTA TCA AGC CCT-3′, reverse: 5′-ACA AGC CTT TGC TCC AGA CCC TAT-3′) , mouse IL-1β (forward: 5′-TGG AAA AGC GGT TTG TCT -3′, reverse: 5′- ATA AAT AGG TAA GTG GTT GCC -3′) and mouse IL-18 (forward: 5′- TGG TTC CAT GCT TTC TGG ACT CCT -3′, reverse: 5′- TTC CTG GGC CAA GAG GAA GTG ATT -3′) were procured from Sigma. For real time PCR, cDNA was synthesised using Advantage RT-for-PCR kit (Clontech laboratories, CA) and 500 ng of cDNA was used as a template on ABI Prism 7700 sequence detection system (Applied Biosystems, Foster City, CA). Power SYBR Green PCR master mix (Applied Biosystems) was used for the experiment. The conditions used for real time PCR have been described earlier [50], [52] and were as follows: 95°C for 3 min (1 cycle), 94°C for 20 s, 55°C for 30 s, and 72°C for 40 s (40 cycles). The dissociation curves were generated to check for the specificity of primer annealing to the template. The real time PCR results were then normalized to 18S rRNA internal control and quantified using comparative C_t_ method (2^−[Δ][Δ]Ct^) [56] and analyzed using the iCycler Thermal Cycler Software (Applied Biosystems).

### Small interfering RNA (siRNA) transfection: Knockdown experiments

ON-TARGET plus short interfering RNA (SiRNA) against mouse NLRP3 (target: 5′-GGU GAA AUG UAC UUA AAU C - 3′) was ordered from the predefined SiRNA library of Dharmacon RNAi technologies (Thermo fisher Scientific, USA). Scrambled SiRNA (ScRNA) (sense: 5′- GUG CAC AUG AGU GAG AU UU- 3′) was designed using an online SiRNA design software (Ambion, Applied biosystems, Austin, USA) and was synthesized by a Dharmacon RNAi technologies (Thermo fisher Scientific, USA). 100 nM of NLRP3 SiRNA was used for transfection using Lipofectamine RNAi max (Invitrogen, Carlsbad, CA, USA) according to the manufacturer's protocol. Briefly, BV-2 cells were seeded and maintained in sets of three at 37°C and 5% CO2 and when the cells were 70% to 80% confluent, they were transfected in Opti-MEM for 6 hours after which fresh 10% DMEM was added to the cells for 30 hours. The cells were then treated with 5 MOI of JEV for 1.5 h followed by incubation in serum free media for different time points. Mock-infected control group received only lipofectamine treatment. mRNA and protein were isolated from these cells to analyse the levels of NLRP3, IL-1β and IL-18 as well as caspase-1 activity.

### Caspase-1 assay

Caspase-1 activation assay was performed using caspase-1 assay kit (Promega, WI, USA), as per the manufacturer's protocol. Briefly, duplicate wells of a flat bottom, black polystyrene 96 well plate (Nunc, USA) were coated with blank, assay and negative control reaction mixtures, prepared according to the manufacturer's protocol. The assay mixture contained caspase Assay buffer, DMSO (Sigma Aldrich), 100 mM DTT (Sigma Aldrich), 50 µl treated cell extract and deionized H_2_O. The negative control additionally consisted of 2.5 mM caspase-1 inhibitor (Ac-YVAD-CHO) apart from the above mentioned reagents. The blank reaction did not contain the cell extract. The AMC standard was prepared simultaneously at various different dilutions according to the procedure mentioned in the assay kit. Plate was covered with parafilm and incubated at 30°C for 30 minutes. 2.5 mM of substrate (Ac-YVAD-AMC) was added to all the wells and incubated again at 30°C for additional 1 h. The fluorescence of the reactions was measured at an excitation wavelength of 360 nm and emission wavelength of 460 nm using the spectroflurometer (Varioskan Flash multimode reader, Thermo Electron Corporation, Finland). The concentrations of the samples were calculated using the AMC standard reference curve. For caspase-1 inhibition experiments, BV-2 cells incubated with 5 µM caspase-1 inhibitor Z-YVAD-FMK for 30 min prior to JEV infection.

### Cytokine Bead Array (CBA)

The CBA kit (BD Biosciences, NJ, USA) was used to quantitatively measure cytokine levels in the mock-infected control and JEV infected BV-2 cells. Using 50 µl of mouse inflammation standard and sample dilutions, the assay was performed according to the manufacturer's instructions and analyzed on the FACS Calibur (Becton Dickinson). This method quantifies soluble particles, in this case cytokines using a fluorescence based detection mechanism. The beads, coated with IL-6, TNF-α and CCL2 react with test lysates and standards, to which fluorescence dyes are then added. Analysis was performed using CBA software that allows the calculation of cytokine concentrations in unknown lysates [35].

### Statistical analysis

Data are represented as the mean ± standard error of the mean (SEM) from at least three independent experiments. The data generated were analyzed statistically byone way analysis of variance (ANOVA) with Bonferroni's post hoc test using SigmaStat 3.5 software (Systat Software Inc., San Jose, California). A statistical p-value of 0.01 and 0.05 were considered significant.

## Supporting Information

Figure S1 **Replication competent JEV and not host derived factors are responsible for caspase-1 activity.** (**A**) Plaque assay carried out with JEV infected mouse brain homogenates (JEV) along with UV irradiated homogenates of JEV infected mouse brains (UV-JEV). (**B**) Caspase-1 activity measured from BV-2 cells that were treated with mock-treated mice brain homogenates (Ctrl) as well as JEV treated mouse brain homogenates (JEV) and JEV treated mouse brain homogenates that are UV irradiated (UV-JEV). (**C–D**) ELISA study showing the levels of IL-1β (**C**) and IL-18 (**D**) in Ctrl, JEV as well as UV-JEV conditions. Graph represents IL-1β and IL-18 levels in pg/ml. Data represent mean ± SEM from 3 independent experiments performed in duplicate. Statistical differences were evaluated using the one way ANOVA with Bonferroni's post hoc test. **, Statistical difference in comparison to cells treated with control brain homogenate (**p<0.01) and #, Statistical difference with respect to JEV infected condition (p<0.01).
(TIF)
