# Supplementary figures and images for: NLRP3 Inflammasome: Key Mediator of Neuroinflammation in Murine Japanese Encephalitis
Source: PLoS One. 2012 Feb 29;7(2):e32270. doi: 10.1371/journal.pone.0032270 (PMC3290554; doi:10.1371/journal.pone.0032270)

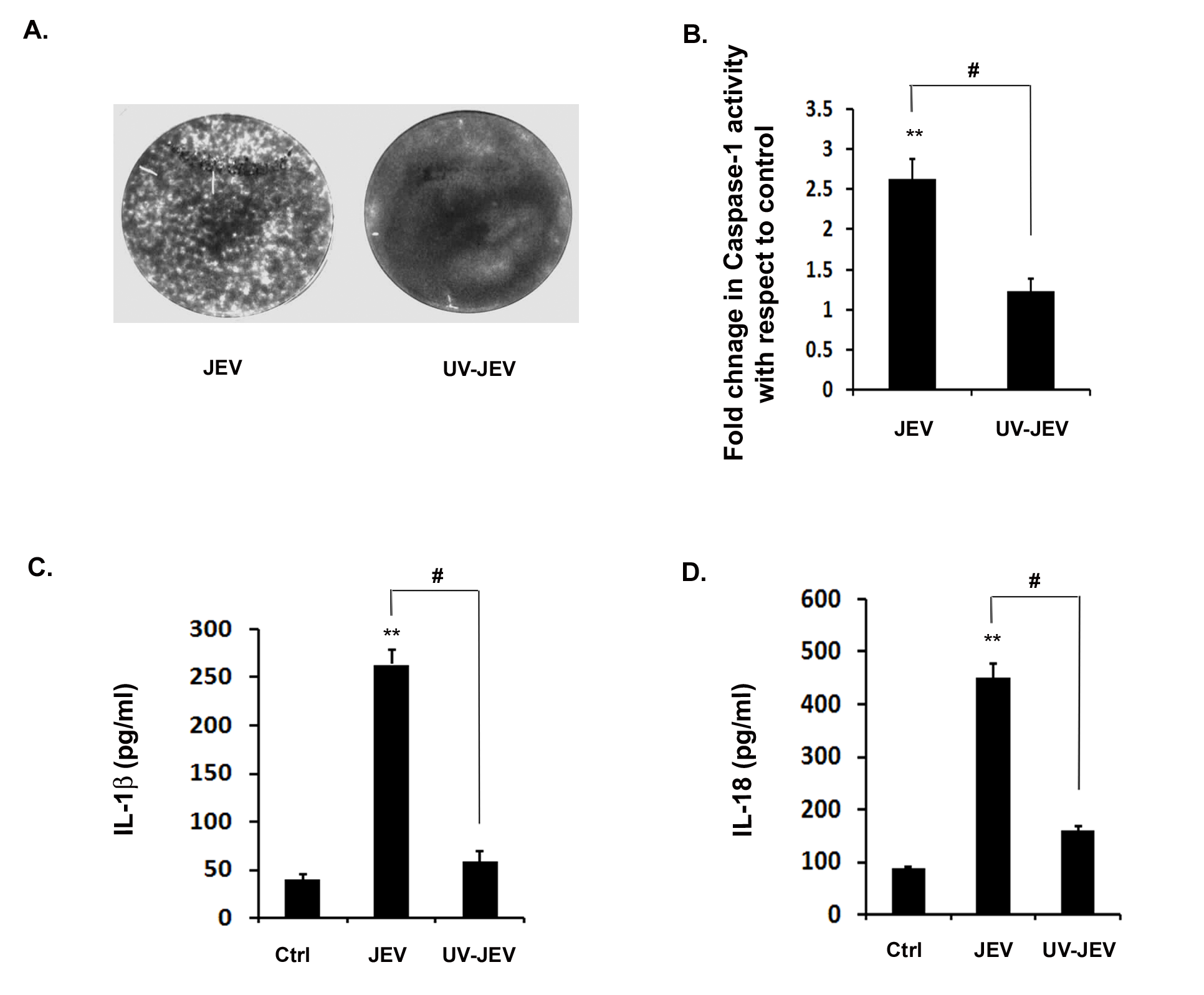

Supplement: Figure S1 — Replication competent JEV and not host derived factors are responsible for caspase-1 activity. (A) Plaque assay carried out with JEV infected mouse brain homogenates (JEV) along with UV irradiated homogenates of JEV infected mouse brains (UV-JEV). (B) Caspase-1 activity measured from BV-2 cells that were treated with mock-treated mice brain homogenates (Ctrl) as well as JEV treated mouse brain homogenates (JEV) and JEV treated mouse brain homogenates that are UV irradiated (UV-JEV). (C–D) ELISA study showing the levels of IL-1β (C) and IL-18 (D) in Ctrl, JEV as well as UV-JEV conditions. Graph represents IL-1β and IL-18 levels in pg/ml. Data represent mean ± SEM from 3 independent experiments performed in duplicate. Statistical differences were evaluated using the one way ANOVA with Bonferroni's post hoc test. **, Statistical difference in comparison to cells treated with control brain homogenate (**p<0.01) and #, Statistical difference with respect to JEV infected condition (p<0.01). (TIF) [file pone.0032270.s001.tif]
